# Hyaluronan modulates accumulation of hypoxia-inducible factor-1 alpha, inducible nitric oxide synthase, and matrix metalloproteinase-3 in the synovium of rat adjuvant-induced arthritis model

**DOI:** 10.1186/ar3365

**Published:** 2011-06-16

**Authors:** Li-Wei Chou, John Wang, Pei-Lin Chang, Yueh-Ling Hsieh

**Affiliations:** 1 Department of Physical Therapy, Graduate Institute of Rehabilitation Science, China Medical University, 91 Hsueh-Shih Road, Taichung, Taiwan 40202, Republic of China; 2 Department of Physical Medicine and Rehabilitation, China Medical University Hospital, 2 Yuh-Der Road, Taichung, Taiwan 40447, Republic of China; 3 School of Chinese Medicine, China Medical University, 91 Hsueh-Shih Road, Taichung, Taiwan 40202, Republic of China; 4 Department of Pathology and Laboratory Medicine, Taichung Veterans General Hospital, 160, Sec. 3, Chung-Kang Road, Taichung, Taiwan 40705, Republic of China; 5 Institute of Biomedical Nutrition, Hungkuang University, 34 Chung-Chie Road, Taichung, Taiwan 40443, Republic of China

## Abstract

**Introduction:**

Hypoxia is a feature of the inflamed synovium in rheumatoid arthritis (RA). Intra-articular injection of hyaluronan (HA) may be considered a potential way to treat RA. However, the exact molecular mechanism of HA on decreased cellular responses to hypoxic environment is unclear. The present study has been designed to use the adjuvant-induced arthritis model to examine the effects of HA on the changes of immunohistochemical expressions of hypoxia-inducible factor-1alpha (HIF-1alpha), inducible nitric oxide synthase (iNOS), and matrix metalloproteinase-3 (MMP3) in the synovial tissues at the early phase of arthritic inflammation.

**Methods:**

Monoarthritis was induced in adult male Sprague-Dawley (250-300 g) via intraarticular injection of complete Freund's adjuvant (CFA) into the tibiotarsal joint. The CFA-induction arthritis animals were divided into three groups: treatment (intraarticular injection of HA), placebo (intraarticular injection of saline) and controls (no treatments). Functional evaluations of edema and pain behavior, histology, and HIF-1alpha, iNOS, and MMP3 immunohistochemistry were performed before, after the first injection, three injections, and on the follow-up injection of the treatments.

**Results:**

Intra-articular injection of HA also significantly suppressed the mechanical allodynia (*p *< 0.001) and overexpressions of HIF-1alpha (*p *< 0.001), iNOS (*p *= 0.004) and MMP3 (*p *< 0.001) immunoreactivity in synovium.

**Conclusions:**

This study demonstrated that early intervention of HA is an effective protection against accumulation of inflammation-induced HIF-1alpha, iNOS, and MMP3 to limit erosive damage in CFA-induced model of arthritis.

## Introduction

A hypoxic microenvironment is a hallmark of the inflamed synovium and its importance in the pathogenesis of rheumatoid arthritis (RA) has been documented [1-4]. In human and animal arthritis models, the importance of hypoxia for the development and persistence of RA has been demonstrated [1,5]. Previous studies have demonstrated the hypoxic nature of the synovium of patients with RA and the constitutive expression of hypoxia-inducible factor-1-alpha (HIF-1α), a key regulator of hypoxia transcriptional response. In RA joints hypoxia has been shown to express increased amounts of HIF-1α and HIF-1 target genes in synovial lining cells and articular chondrocytes under hypoxic conditions, which aggravate joint inflammation [6,7]. Previous studies also demonstrated that hypoxia takes place in the synovium at the pre-arthritic stage or early stage of the disease and has a close spatial relationship and positive severity correlation with synovitis [8]. Therefore, HIF-1α is identified as a key player in the pathogenesis of RA and a potential therapeutic target in RA development.

Nitric oxide (NO) synthesized from arginine by nitric oxide synthases (NOS) is an important chemical mediator of inflammation. The inducible isoform of NOS (iNOS) is primarily responsible for producing large amounts of NO and its overexpression has been linked to the progressive inflammation and tissue destruction observed in hypoxic experimental arthritis [9,10] and human rheumatoid synovium [11,12]. Matrix metalloproteinases (MMPs), the most important matrix-degrading enzymes in RA, act as key mediators of the resorption of cartilage, bone, synovial fluid, and adjacent soft tissue, and this resorption occurs as part of the pathological destruction of joint tissue [13]. Among dozens of MMPs, MMP3 (stromelysin 1) has been reported to be the major enzyme produced by fibroblasts and macrophage-like cells in the synovium, and the level of MMP3 has been reported to be significantly higher in synovial fluids from patients with RA [14-16]. Under the inflammatory conditions of RA, the levels of HIF-1α, iNOS, and MMP3 are significantly higher in synovial fluids in previous studies and thus are implicated in the pathogenesis of RA. Expressions of iNOS and MMP3 are probably regulated by HIF-1α in the cellular response to hypoxic and inflammatory environments [11,17,18]. Therefore, inhibition or downregulation of these molecules (or both) may exert anti-hypoxic and anti-inflammatory effects.

Hyaluronan (HA) is a polymer of disaccharides and has a high capacity for holding water and possesses high viscoelasticity [11]. The intra-articular supplementation of HA can replace synovial fluid, which has lost its viscoelastic properties. HA has been widely used for the treatment of osteoarthritis (OA) [19]. HA not only is a lubricating agent but its exogenous administration can suppress the expression of inflammatory cytokines, MMPs, and free oxygen radicals to reduce inflammation in a post-laminectomy rat model [20] and patients with RA [21]. Therefore, it has been expected that the intra-articular injection of HA is more efficacious in treating RA, which principally characterizes articular synovitis [21,22]. However, for RA joint treatment, the clinical use of HA is still rare because its immunoregulatory action is still debatable.

Complete Freund's adjuvant (CFA)-induced arthritis shares some characteristics of RA. This model mirrors much of the pathology of RA, including hyperplasia of the synovial tissues, inflammatory infiltration of the joints, and destruction of bone and cartilage in the synovial joint [23]. The present study has been designed to use the adjuvant-induced arthritis model to examine the effects of HA on the changes of immunohistochemical expressions of HIF-1α, iNOS, and MMP3 in the synovial tissues in the early phase of arthritic inflammation. We hypothesize that the addition of HA will alleviate inflammatory nociception and impede the accumulation of arthritis-induced HIF-1α, iNOS, and MMP3 production in the early phase of the experimental arthritic inflammatory joint. This hypothesis, if correct, will offer at least a partial explanation for the efficacy of topical HA application in the subsequent inhibition of hypoxic inflammation in this preclinical model.

## Materials and methods

### General design

**Figure 1:**
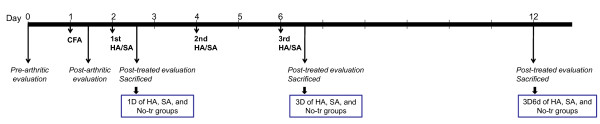
**Experimental design of the sequence of events for the entire course of the experiment**. After evaluations that included measurements of paw edematous swelling and pain threshold, the animals were sacrificed for histology and immunohistochemistry. 1D, one dose; 3D, three doses; 3D6d, follow-up at the 6th day after three doses; CFA, complete Freund's adjuvant; HA, hyaluronan; No-tr, no treatment; SA, saline.

Arthritis was induced in all animals by intra-articular injection of CFA. After a day of CFA induction, the arthritic animals were randomly divided into one of three groups (n = 30 per group) according to the treatment administered: (a) the 'no treatment' (No-tr) group, which consisted of controls that received a sham injection by needling (that is, no solution was administered); (b) the SA, or placebo, group, which received 50 μL of saline; and (c) the HA group, which received 50 μL of HA. Injections for all three groups were intra-articularly administered. Injections of HA or saline were given every 2 days (days 2, 4, and 6). The evaluation instruments were edematous swelling of the paw, pain behavioral assessments, histology, and immunohistochemistry. Assessments were performed at day 0 (pre-arthritic), day 1 (post-arthritic), 3 hours after the treatment of one injection (one dose, 1D) on day 2, after three injections (three doses, 3D) on day 6, and 6 days after three doses (3D6d) on day 12. A flow diagram is presented in Figure 1.

### Animal preparation

Ninety adult male Sprague-Dawley (SD; purchased from BioLASCO Taiwan Co., Ltd., Taiwan, Republic of China) rats weighing 250 to 300 g were kept in the Laboratory Animal Center of China Medical University. An effort was made to minimize discomfort and to reduce the number of animals used. All animal experiments were conducted with the approval of the Animal Care and Use Committee of China Medical University in accordance with the Guidelines for Animal Experimentation.

### Induction of monoarthritis

Monoarthritis was induced by an injection of CFA into the unilateral ankle articular cavity. The rats were briefly anesthetized with 4% isoflurane (AERRANE; Baxter Healthcare Corporation, San Juan, Puerto Rico). A 28-gauge needle was vertically inserted distally into the articular cavity from the gap between the tibiofibular and tarsus bone. CFA with a volume of 50 μL (10 mg of mycobacterium, F5881; Sigma-Aldrich, St. Louis, MO, USA) was then injected. The monoarthritic animals were placed separately in clear acrylic containers (10.5-inch width × 19-inch diameter × 8-inch height), and free movement was allowed for at least 24 hours to let the animals adjust to these conditions before any experimentation was performed.

### Ultrasound-guided hyaluronan injection

While the animals were under brief isoflurane anesthesia, ultrasound (Terason t3000™ Ultrasound System; Terason Division, Teratech Corporation, Burlington, MA, USA)-guided injection was performed on the lateral side of the tibiotarsal joint, and the transducer in the sagittal plane showed the distal end of the tibia and proximal part of the tarsus in the image plane. Needle insertion was performed perpendicularly to the transducer. HA injection (molecular weight of 1.2 to 1.4 × 10^6 ^Da; Ostenil^®^, 10 mg/mL sodium hyaluronate; TRB Chemedica AG, München, Germany) was documented by recording an image clip during injection with the needle tip in the image plane.

### Pain threshold assessment

The pain thresholds were determined by nociceptive thresholds to mechanical stimulation. The test consisted of evoking a hind paw flexion reflex with a handheld force transducer (electronic von Frey anesthesiometer; IITC Inc., Woodland Hills, CA, USA) adapted with a 0.5 mm^2 ^polypropylene tip. In a quiet room, the rats were placed in acrylic cages (32 × 22 × 27 cm high) with a wire grid floor for 15 to 30 minutes of habituation prior to testing. The polypropylene tip was applied perpendicularly to the central area of the hind paw with sufficient force to bend the filaments into an 'S' shape for 3 to 4 seconds. The test consisted of poking a hind paw to provoke a flexion reflex followed by a clear flinch response after paw withdrawal. Testing was initiated with the filament corresponding to 20 log of force (g). The filaments were applied with a gradual increase in pressure until a withdrawal reflex response was finally detected from the animal. The response to this filament is defined if a series of weaker or stronger filaments would be tested. The weakest filament able to elicit a response was taken to be the paw withdrawal threshold (g). The intensity of the pressure was recorded, and the final value for the response was obtained by averaging five measurements.

### Measurement of edematous swelling of the paw

The extent of peripheral swelling was assessed by measuring the circumference of the paw at intact and CFA-injected sites with a flexible tape. The paw circumference was obtained by averaging three measurements. The difference in the ankle circumference between the initial value (pre-arthritic data) and that at each time point after injection is expressed as change (percentage) = 100% × [(post-arthritic circumference)-(pre-arthritic circumference)]/(pre-arthritic circumference). All assessments, including paw withdrawal and swelling measurements, were performed with the assessor blinded with respect to treatment.

### Histology and immunohistochemistry

Animals were killed by anesthetic overdose after treatments of 1D (n = 10 for each group), 3D (n = 10 for each group), and 3D6d (n = 10 for each group) on days 2, 6, and 12. Hind ankles were collected for histological and immunohistological analysis. The formalin-fixed, paraffin-embedded joint tissues (including synovium and cartilage tissues) were cut at a thickness of 5 μm for histology and immunohistochemistry. Histological confirmation of the arthritic pathology was performed with hematoxylin and eosin-stained sections. Sections were deparaffinized in 200 mL of Trilogy (Cell Marque Corporation, Rocklin, CA, USA) and incubated with 3% H_2_O_2 _in methanol for 20 minutes at room temperature. Subsequently, sections were treated with proteinase K (Sigma-Aldrich) at 0.1 mg/mL for 20 minutes at room temperature to unmask epitopes and this was followed by phosphate-buffered saline (PBS) rinse. Sections were incubated with blocking buffer (Power Block™; Biogenex, Fremont, CA, USA) for 2 hours at room temperature followed by incubation overnight at 4°C with the mouse monoclonal antibody anti-HIF-1α (diluted 1:100; Thermo, Fremont, CA, USA) and with the following rabbit polyclonal antibodies: anti-iNOS (diluted 1:200; Thermo) and anti-MMP3 (diluted 1:200; Abbiotec, San Diego, CA, USA). After three washes with PBS containing 0.05% Tween-20 for 10 minutes, sections were incubated with biotinylated anti-rabbit and anti-mouse immunoglobulins (Jackson ImmunoResearch Laboratories, Inc., West Grove, PA, USA) followed by a 30-minute peroxidase-conjugated streptavidin incubation (Jackson ImmunoResearch Laboratories, Inc.). Sections were incubated with 3,3'-diaminobenzidine (Biogenex), dehydrated, and cover-slipped with Permount (Sigma-Aldrich, St. Louis, MO, USA). Negative controls were performed by substituting the primary antibody with non-immune serum.

The histopathology of synovium was analyzed by the non-parametric scoring system described by Smith and colleagues [24]. The scores ranged from 0 to 3 for each of the tissue criteria, including intimal hyperplasia, lymphocytic infiltration, subintimal fibrosis, and vascularity. The higher aggregate score was considered to reflect increased pathological changes. Five randomly selected sections were scored and repeated two times for statistical analysis. Quantitative analysis of immunostainings was carried out by light microscopy in synovial tissue lining the joint cavity and synovial tissue attached to the cartilage. The number of HIF-1α, iNOS, and MMP3 immunoreactive cells was counted among at least five alternate sections in the more representative fields by using a microscope. Positive nuclei and cytoplasm staining cells for HIF-1α, iNOS, and MMP3 were counted in high-power fields (× 200 magnification) that contained synovial lining cells. The area sizes of high-power fields were calculated by using a stage micrometer (with 100 gradations of 0.01 mm each) when viewed using a × 200 objective. Ten fields of each slide were counted for all samples and repeated three times for statistical analysis. Results were expressed as the proportion (percentage) of labeled cells per square millimeter of synovium. For statistical analysis, the mean value obtained from the repeated counts was used. All of the scoring and quantitative analyses were assessed by two independent observers who were blinded to the origin of the sections to avoid bias from interobserver variability.

### Statistical analysis

The differences of value in each assessment between pre- and post-arthritic evaluations were analyzed by Student *t *test. The differences among the HA, SA, and No-tr groups on each dosage (1D, 3D, and 3D6d) were analyzed using analysis of variance and later were analyzed further by a Bonferroni *post hoc *method. Similar statistical analysis methods were used to test the differences among dosages in each group. Non-parametric data (histological synovial scoring) were analyzed using the Kruskal-Wallis test for multiple groups and Mann-Whitney *U *tests for between-group comparisons. The Pearson correlation test was applied to study the correlations between pain withdrawal threshold and expressions of immunoreactivities. A *P *value of less than 0.05 was considered statistically significant. All data were analyzed using SPSS version 10.0 for Windows (SPSS Inc., Chicago, IL, USA).

## Results

### Effect of hyaluronan on complete Freund's adjuvant-induced edema

**Figure 2:**
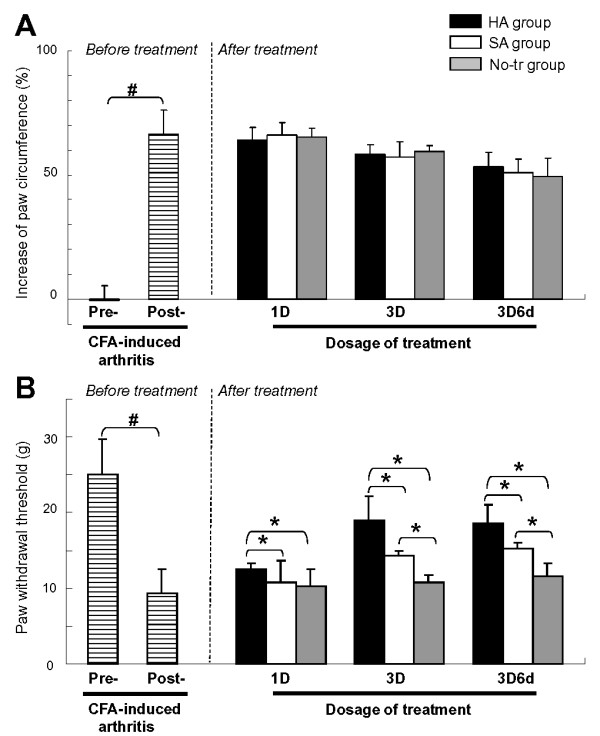
**Results of edema (a) and pain behavioral (b) assessments**. Data were calculated before treatment at the conditions of pre-complete Freund's adjuvant (CFA)-induced and post-CFA-induced arthritis and after treatment at conditions of one dose (1D), three doses (3D), and follow-up at the 6th day after three doses (3D6d) in hyaluronan (HA), saline (SA), and 'no treatment' (No-tr) groups. Each bar represents the mean ± standard deviation in body weight and mean ± standard error of the mean in paw circumference and withdrawal threshold. ^#^*P <*0.05, Student *t *test for comparison of pre- and post-arthritic conditions before treatment. *P <0.05, Bonferroni *post hoc *test for comparison of difference between groups at dosages of 1D, 3D, and 3D6d after treatment.

The serial alterations of the percentage of edema (mean ± standard error of the mean, or SEM) throughout the whole experiment for each group are shown in Figure 2A. After a day of CFA induction, all animals developed severe monoarthritis in the injected paw. There were no significant differences in the non-injected intact paw on circumference among pre- and post-arthritic and post-treatment conditions for each group (*P >*0.05, data not shown). The edema of the CFA-injected paw gradually increased, reaching a maximal swelling of 65.51%, whereas there were significant differences in edema between pre- and post-arthritic conditions (*P <*0.001).

After treatment, the significant time-dependent differences in edema development were observed in each group (HA group: *P <*0.001; SA group: *P <*0.001; and No-tr group: *P <*0.001). However, there was no difference in the edema of arthritic paws among the HA, SA, and No-tr groups after treatments of 1D (*P *= 0.22), 3D (*P *= 0.41), and 3D6d (*P *= 0.31). Therefore, intra-articular injections of HA, regardless of different dosages for 1D, 3D, and 3D6d, did not ameliorate joint swelling compared with either the SA or the No-tr group.

### Effect of hyaluronan on complete Freund's adjuvant-induced inflammatory mechanical nociception

The serial alterations of the paw withdrawal threshold (mean ± SEM) throughout the whole experiment for each group are shown in Figure 2B. The mean threshold was 25.07 ± 4.68 g at pre-arthritic conditions. However, after CFA induction, it decreased to 9.32 ± 3.16 g. There was a significant difference with pre-arthritic conditions (*P <*0.001).

The significant differences in paw withdrawal threshold were shown among the HA, SA, and No-tr groups after treatment of 1D (*P *= 0.008), 3D (*P <*0.001), and 3D6d (*P <*0.001). A significantly lower threshold existed after treatment of 1D, 3D, and 3D6d in the SA and No-tr groups compared with those in the HA group (HA versus SA, *P *= 0.04; HA versus No-tr, *P *= 0.01 for 1D; HA versus SA, *P *< 0.001; HA versus No-tr, *P <*0.001 for 3D; HA versus SA, *P <*0.001; HA versus No-tr, *P *= 0.001 for 3D6d). The analysis also showed that there was a significantly lower threshold in the No-tr group compared with the SA group after treatment of 3D (*P = *0.03) and 3D6d (*P = *0.01). However, no significant difference was observed between these groups after treatment of 1D (*P = *1.0).

There were significant differences among three dosages in the HA group (*P <*0.001) but not in the SA (*P *= 0.84) and No-tr (*P *= 0.56) groups. After HA treatment, the paw withdrawal threshold showed a significant increase in 3D and 3D6d treatments compared with 1D treatment (1D versus 3D, *P <*0.001; 1D versus 3D6d, *P <*0.001). However, no difference was observed between the 3D and 3D6d of HA treatments (*P *= 0.05).

### Histopathological assessments

**Figure 3:**
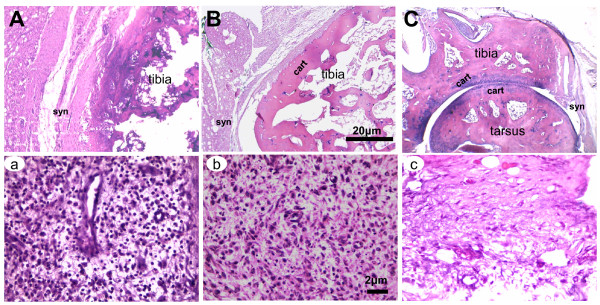
**Histopathology of arthritis joints**. Representative hematoxylin and eosin sections of hind paws obtained from adjuvant-induced arthritic animals treated with intra-articular injections of 'no treatment' (No-tr) **(A)**, saline (SA) **(B)**, and hyaluronan (HA) **(C)**. In rats in the No-tr group, in which cartilaginous tissue could not be clearly detected, bone damage was even greater and massive inflammatory cells infiltrated the synovium **(a)**. Similar changes were observed in rats treated with SA. Cartilage erosion was more pronounced and the extensively expanded synovial pannus was more densely infiltrated with mononuclear cells **(b)**. In rats treated with HA, the joints were much less inflamed, and lymphocyte accumulation **(c) **and cartilage damage decreased. There was no sign of bone destruction. 1D, one dose; 3D, three doses; 3D6d, follow-up at the 6th day after three doses; cart, cartilage; syn, synovial tissue.

Widening of the synovial cavity, infiltration of inflammatory cells, thickening of the synovial membrane, narrowing of the synovial space, disruption of the cartilaginous tissue, and bone erosion were apparent in control rats of the No-tr group (Figure 3A, 3a) and SA group (Figure 3B, 3b). The tibiotarsal joints of rats treated with 1D, 3D, and 3D6d of HA were less inflamed, as revealed by a decreased number of inflammatory cells, synovial membrane thickening, and cartilage destruction (Figure 3C, 3c). There were significant differences in lymphocytic infiltration and aggregate score of non-parametric criteria observed among ankle joint synovium from the HA, SA, and No-tr groups treated with 1D, 3D, and 3D6d (*P *< 0.05) (Table 1). Lymphocytic infiltrations in synovium were significantly reduced after HA treatment when compared with those treated with SA or No-tr (HA versus SA, HA versus No-tr, *P *< 0.05 in all doses). There were no significant differences in intimal hyperplasia, subintimal fibrosis, and vascularity among the three groups (*P *> 0.05).

**Table 1:** Results of histopathological scores of synovium for sections in arthritic ankle joint sampled from three treatment groups

Dose	Group	Intimal hyperplasia	Subintimal fibrosis	Lymphocytic infiltration	Vascularity	Aggregate score
1D	HA	2.45 ± 0.11	2.60 ± 0.11	1.50 ± 0.11^a, b^	2.05 ± 0.11	7.80 ± 0.26^a, b^
	SA	2.60 ± 0.11	2.60 ± 0.11	2.50 ± 0.11	2.10 ± 0.12	9.10 ± 0.31
	No-tr	2.65 ± 0.11	2.65 ± 0.10	2.95 ± 0.05	2.20 ± 0.12	9.75 ± 0.24
	^c^*P *value among groups	*P *> 0.05	*P *> 0.05	*P <*0.001	*P *> 0.05	*P <*0.001
3D	HA	2.50 ± 0.11	2.70 ± 0.11	1.40 ± 0.13^a, b^	2.20 ± 0.09	8.05 ± 0.31^a, b^
	SA	2.80 ± 0.09	2.70 ± 0.10	2.55 ± 0.11	2.15 ± 0.11	9.55 ± 0.28
	No-tr	2.80 ± 0.09	2.70 ± 0.11	2.85 ± 0.08	2.20 ± 0.14	9.95 ± 0.32
	^c^*P *value among groups	*P *> 0.05	*P *> 0.05	*P <*0.001	*P *> 0.05	*P <*0.001
3D6d	HA	2.50 ± 0.11	2.50 ± 0.11	1.40 ± 0.11^a, b^	2.15 ± 0.11	7.85 ± 0.25^a, b^
	SA	2.70 ± 0.11	2.60 ± 0.10	2.77 ± 0.10	2.20 ± 0.14	9.6 ± 0.36
	No-tr	2.70 ± 0.11	2.70 ± 0.11	2.85 ± 0.08	2.40 ± 0.11	10.05 ± 0.33

Sections were stained with hematoxylin and eosin. Values are presented as mean ± standard error of the mean. ^a^*P *< 0.05, showed statistically significant differences between hyaluronan (HA) and saline (SA) groups; ^b^*P *< 0.05, showed statistically significant differences between HA and 'no treatment' (No-tr) groups; Mann-Whitney *U *ranked tests were used for between-group comparisons. ^c^Tested with Kruskal-Wallis test. 1D, one dose; 3D, three doses; 3D6d, follow-up at the 6th day after three doses.

### Immunohistochemical assessments on location of HIF-1α, iNOS, and MMP3

**Figure 4:**
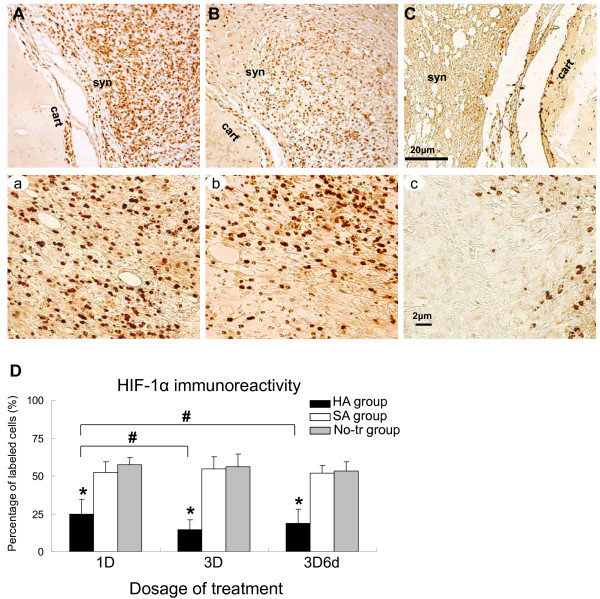
**Representative immunohistochemical sections of hypoxia-inducible factor-1-alpha (HIF-1α) immunoreactivity**. Sections obtained from the arthritic synovium treated with intra-articular injections of 'no treatment' (No-tr) **(A)**, saline (SA) **(B)**, and hyaluronan (HA) **(C)**. At higher-power magnification, it is evident that these positive (brown staining) immunoreactivities were clearly localized in both the nucleus and cytoplasm of arthritic synovium in the sections from No-tr **(a) **and SA **(b) **animals. Administration of HA **(c) **to adjuvant-induced rat produced a marked reduction in the immunostaining for HIF-1α. Quantitative analysis **(D) **of positive-labeled cells in synovium for HIF-1α immunohistochemistry at the early phase of inflammation of each group was presented in the average proportion of labeled neurons (mean ± standard error of the mean). *P <0.05, showed significant differences between groups when either SA or No-tr is compared with HA group using Bonferroni *post hoc *test. Significant differences were found between HA versus SA groups and HA versus No-tr groups. ^#^*P <*0.05, showed significant differences between dosages tested by Bonferroni *post hoc *test. 1D, one dose; 3D, three doses; 3D6d, follow-up at the 6th day after three doses; cart, cartilage; syn, synovial tissue.

**Figure 5:**
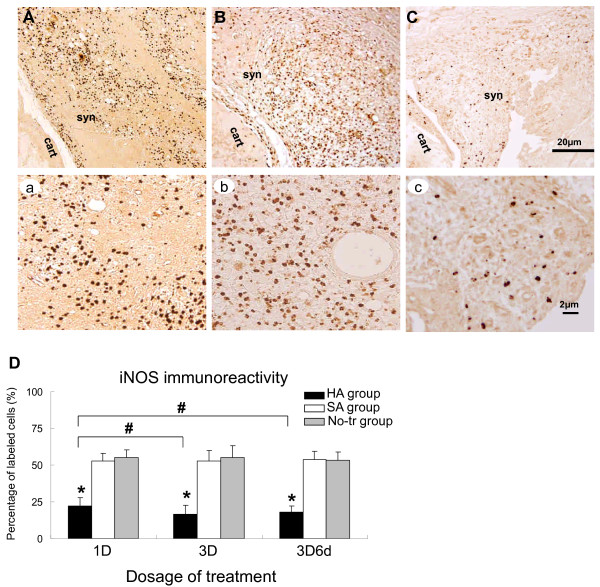
**Representative immunohistochemical sections of inducible nitric oxide synthase (iNOS) immunoreactivity**. Sections obtained from the arthritic synovium treated with intra-articular injections of 'no treatment' (No-tr) **(A)**, saline (SA) **(B)**, and hyaluronan (HA) **(C)**. At higher-power magnification, it is evident that these positive (brown staining) immunoreactivities were clearly localized in both the nucleus and cytoplasm of arthritic synovium in the sections from No-tr **(a) **and SA **(b) **animals. Administration of HA **(c) **to adjuvant-induced rat produced a marked reduction in the immunostaining for iNOS. Quantitative analysis **(D) **of positive-labeled cells in synovium for iNOS immunohistochemistry at the early phase of inflammation of each group was presented in the average proportion of labeled neurons (mean ± standard error of the mean). *P <0.05, showed significant differences between groups when either SA or No-tr is compared with HA group using Bonferroni *post hoc *test. Significant differences were found between HA versus SA groups and HA versus No-tr groups. ^#^*P <*0.05, showed significant differences between dosages tested by Bonferroni *post hoc *test. 1D, one dose; 3D, three doses; 3D6d, follow-up at the 6th day after three doses; cart, cartilage; syn, synovial tissue.

**Figure 6:**
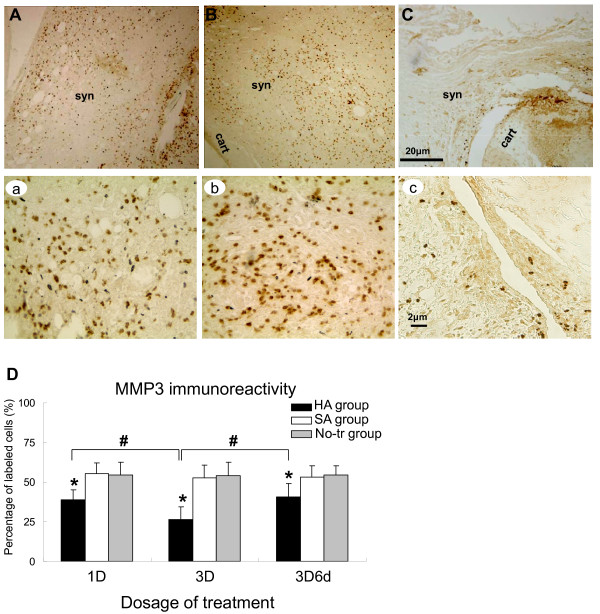
**Representative immunohistochemical sections of matrix metalloproteinase-3 (MMP3) immunoreactivity**. Sections obtained from the arthritic synovium treated with intra-articular injections of 'no treatment' (No-tr) **(A)**, saline (SA) **(B)**, and hyaluronan (HA) **(C)**. At higher-power magnification, it is evident that these positive (brown staining) immunoreactivities were clearly localized in both the nucleus and cytoplasm of arthritic synovium in the sections from No-tr **(a) **and SA **(b) **animals. Administration of HA **(c) **to adjuvant-induced rat produced a marked reduction in the immunostaining for inducible nitric oxide synthase. Quantitative analysis **(D) **of positive-labeled cells in synovium for MMP3 immunohistochemistry at the early phase of inflammation of each group was presented in the average proportion of labeled neurons (mean ± standard error of the mean). *P <0.05, showed significant differences between groups when either SA or No-tr is compared with HA group using Bonferroni *post hoc *test. Significant differences were found between HA versus SA groups and HA versus No-tr groups. ^#^*P <*0.05, showed significant differences between dosages tested by Bonferroni *post hoc *test. 1D, one dose; 3D, three doses; 3D6d, follow-up at the 6th day after three doses; cart, cartilage; syn, synovial tissue.

Overexpressions of HIF-1α, iNOS, and MMP3 immunoreactivities were found within the synovial tissue in the No-tr group (Figures 4A, 5A, and 6A) and the SA group (Figures 4B, 5B, and 6B). At higher-power magnification, it is evident that these positive immunoreactivities were clearly localized in both the nucleus and cytoplasm of arthritic synovium (Figures 4a, 5a, 6a, 4b, 5b, and 6b). The primary cells exhibiting specific HIF-1α, iNOS, and MMP3 immunoreactivities were morphologically consistent with macrophages, mainly in inflammatory infiltrate and invasive pannus of the inflamed synovial joint. Synovial lining cells and some chondrocytes were also found to be positive for HIF-1α, iNOS, and MMP3. After treatment with HA, the HIF-1α, iNOS, and MMP3 immunoreactivities were reduced (Figures 4C, 5C, and 6C) concurrently with reduced immunoreactivities localized in both the nucleus and cytoplasm of arthritic synovium at higher-power magnification (Figures 4c, 5c, and 6c).

### Quantitative analysis of extent of HIF-1α, iNOS, and MMP3 immunoreactive expressions

After treatment, the significant differences in extent of HIF-1α, iNOS, and MMP3 immunoreactive expressions were shown among the HA, SA, and No-tr groups after treatment of 1D (HIF-1α: *P <*0.001; iNOS: *P <*0.001; MMP3: *P <*0.001), 3D (HIF-1α: *P <*0.001; iNOS: *P <*0.001; MMP3: *P <*0.001), and 3D6d (HIF-1α: *P <*0.001; iNOS: *P <*0.001; MMP3: *P <*0.001). Significantly lower expressions of HIF-1α, iNOS, and MMP3 immunoreactivities existed after treatment of 1D in the HA group-HIF-1α: HA versus SA, *P <*0.001; HA versus No-tr, *P <*0.001 (Figure 4D); iNOS: HA versus SA, *P <*0.001; HA versus No-tr, *P <*0.001 (Figure 5D); MMP3: HA versus SA, *P <*0.001; HA versus No-tr, *P <*0.001 (Figure 6D). The analysis also showed that there were no significant differences in HIF-1α, iNOS, and MMP3 immunoreactivities between the SA and No-tr groups for 1D dosage (HIF-1α: SA versus No-tr, *P *= 0.14; iNOS: *P *= 0.45; MMP3: *P *= 1.0) (Figures 4D, 5D, and 6D). Similar results were found for HIF-1α, iNOS, and MMP3 immunoreactivities for treatments of 3D and 3D6d (Figures 4D, 5D, and 6D).

Significant differences in extent of HIF-1α, iNOS, and MMP3 immunoreactive expressions were shown among 1D, 3D, and 3D6d dosages in the HA group (HIF-1α: *P <*0.001; iNOS: *P *= 0.004; MMP3: *P <*0.001) but not in the SA group (HIF-1α: *P *= 0.56; iNOS: *P *= 0.85; MMP3: *P *= 0.81) or the No-tr group (HIF-1α: *P *= 0.16; iNOS: *P *= 0.50; MMP3: *P *= 0.99). After 3D and 3D6d of HA treatment, the extent of HIF-1α and iNOS immunoreactive expressions significantly reached maximum reduction compared with those of 1D treatment-HIF-1α: 3D versus 1D, *P <*0.001; 3D6d versus 1D, *P = *0.03 (Figure 4D); iNOS: 3D versus 1D, *P *= 0.01; 3D6d versus 1D, *P *= 0.03 (Figure 5D). However, no difference was exhibited between the 3D and 3D6d of HA treatments (HIF-1α: 3D versus 3D6d, *P *= 0.15; iNOS: 3D versus 3D6d, *P *= 1.0). For expression of MMP3 immunoreactivity, significant reduction was found after 3D treatment (3D versus 1D, *P *= 0.001; 3D versus 3D6d, *P <*0.001) (Figure 6D). However, the expression of MMP3 immunoreactivity recovered after 3D6d treatment (3D6d versus 1D, *P *= 1.0).

### Association of pain withdrawal threshold with immunoreactivity results

A significant linear correlation was found between pain withdrawal threshold and immunoreactivity of HIF-1α, iNOS, and MMP3 (Pearson correlation coefficients, *P *< 0.05) (Table 2). There were strong negative associations of pain withdrawal threshold with HIF-1α, iNOS, and MMP3 after 3D treatment and with HIF-1α and MMP3 after 3D6d treatment (0.75 < | Pearson γ | < 1).

**Table 2:** Association of pain withdrawal threshold with the immunoreactivity results given as gamma values

	Pain withdrawal threshold		
	1D	3D	3D6d
HIF-1α	-0.378^a^	-0.848^a^	-0.869^a^
iNOS	-0.280^b^	-0.782^a^	-0.765^a^
MMP3	-0.420^a^	-0.823^a^	-0.856^a^

Correlations were analyzed by Pearson correlation coefficients. ^a^*P *< 0.01; ^b^*P *< 0.05.

1D, one dose; 3D, three doses; 3D6d, follow-up at the 6th day after three doses; HIF-1α, hypoxia-inducible factor-1-alpha; iNOS, inducible nitric oxide synthase; MMP3, matrix metalloproteinase-3.

## Discussion

The results of this study demonstrate that lymphocytic/plasmocytic infiltration in the synovium and accumulation of HIF-1α, iNOS, and MMP3 were suppressed after intra-articular administration of HA at the early phase of adjuvant-induced inflammation. The extent of HIF-1α, iNOS, and MMP3 immunoreactivities was consistent with the results of pain behavioral assessment, which demonstrated an elevation of the mechanonociceptive threshold after administration of HA. To the best of our knowledge, these findings have never been reported by other researchers.

In this model, the early phases of adjuvant-induced arthritis seem to be characterized by acute cytokine-induced inflammation [25]. Owing to infiltration of the injured tissues by immune cells and responses, swelling is a major sign during acute inflammation and might also be considered an important parameter on evaluation of the potential anti-inflammatory effects of compounds [26]. However, as shown in the results of our study, the levels of edematous swelling were not changed after HA treatment in acute inflammation at the early phase of adjuvant-induced arthritis, suggesting the weaker activity against edema of HA in an acute inflammatory animal model. This result is consistent with the animal study with collagen-induced arthritis [27] and human study with OA [28]. The reason is probably due to a HA-induced swelling adverse effect at the injection site occurring. Previous studies revealed that HA may act as either a primary irritant or an inflammatory mediator to induce acute adverse events characterized by transient swelling of the injected joint in some patients [28-30]. The prevalence of adverse effects was noted in 47% of patients after HA supplementation and in 22% of patients treated with saline injections [31]. In this study, the observation time of edema measurement started 3 hours after HA administration, when an adverse effect of a transient increase in swelling at the injection site occurred. Therefore, further study with long-term observations of joint swelling after ceasing HA was needed to clarify the effect of exogenous HA on resolving RA-induced joint edema.

It has been well established by animal behavioral and human clinical studies that elastoviscous solutions of HA could have an analgesic effect when injected into arthritic joints and if appropriately applied to patients with acute arthritic pain [32]. There was significantly less bradykinin found in the crystal-induced arthritic joint after injection of HA [33]. Electrophysiological studies also demonstrated that the rates of neural discharges of the nociceptive afferent fibers innervating the synovial tissue were significantly attenuated and reached a constant rate 2 to 3 hours after injection [32,34,35]. Treatment of HA showed an analgesic effect after the onset of cartilage destruction and pain in a rabbit OA model [36]. To the best of our knowledge, our behavioral study is the first report on the analgesic effect of HA at decreased mechanical allodynia in a rat RA model and is consistent with the findings of previous studies. The intra-articular injection of HA also resulted in elevation of the mechanonociceptive threshold, which was in accordance with the results of immunohistochemistry in this study. HA has been demonstrated to possess a therapeutic effect on OA and this effect has been studied by many researchers. Macroscopic and microscopic evaluations revealed that HA has chondroprotective effects in a rabbit model of OA [37]. Our results showed that HA reduced pathohistological signs, including the degree of infiltration of the synovial membrane by plasma and lymphocytes, in collagen-induced arthritis animals, and are consistent with findings from a previous study [38]. The tendency for decreased cellular infiltration during the early phase of arthritis supports the assumption that HA provides a temporary protective barrier over the cartilage and thereby protects it against CFA insults. HA has also been shown to significantly suppress NO production and inhibit IL-1β-induced MMP3 production from OA synovial tissue *in vitro *and *in vivo *[39-42]. As far as we know, few English language studies of the role of HA on suppression of HIF-1α-mediated hypoxic and inflammatory responses have been conducted in OA models. Owing to less inflammation in OA synovial tissue, there is minor HIF-1α expression in these tissues [5]. However, there is higher expression of HIF-1α immunohistochemistry in RA synovial tissues compared with OA tissues because the latter tissue, by nature, is inflammatory and angiogenic in RA [7]. Therefore, HIF-1α has the potential to serve as an anti-rheumatic drug activity biomarker in the clinic and is expected to significantly affect/accelerate the clinical development of treatment for RA.

The possible important role of HIF-1α in RA has been extensively discussed [43,44]. The presence of both hypoxia and inflammatory proteins in RA synovium, which both lead to HIF-1α stabilization and subsequent HIF-1 activation, seems to highlight the important role of HIF-1α [44]. Elevated synovial angiogenesis is a key event during the course of RA. The modulation and blockade of angiogenesis via drug interventions have been shown to contribute to therapeutic efficacy in rat models of arthritis [45]. HIF-1α probably has a crucial involvement in the angiogenic process of synovium in RA by regulation of its target gene, vascular endothelial growth factor (VEGF) [43]. Inhibition of HIF-1α protein expression and VEGF production by SMP-114, a disease-modifying anti-rheumatic drug, has been shown to be of therapeutic benefit in RA [46]. Oral administration of the inhibitor of heat shock protein 90 (Hsp90), which has been shown to potently reduce HIF-1α-related signaling and VEGF production, has also been found to decrease inflammation and cartilage damage in *in vivo *models of RA [47]. Therefore, suppression of HIF-1α may have key implications in the development of novel therapeutic strategies revolutionizing the treatment of RA. Results showed that HA suppressed the adjuvant-induced overexpression of iNOS and MMP3, and this is consistent with findings from previous studies. To the best of our knowledge, our study is the first to report that HA suppresses HIF-1α. This study revealed the reduction of accumulation of HIF-1α expression in the synovium of an adjuvant-induced RA model after intra-articular HA administration. The suppressive effects on accumulation of inflammation-induced HIF-1α, iNOS, and MMP3 expressions in the synovium may be involved in the therapeutic mechanism of HA intervention used in the treatment of RA. Further molecular studies on expressions of VEGF will be needed to fully support the issue of anti-angiogenic effects of HA.

## Conclusions

Suppression of HIF-1α may be one of the major targets of the therapeutic approach in RA. This study demonstrated that early intervention of HA is an effective protection against accumulation of inflammation-induced HIF-1α, iNOS, and MMP3 and might limit the erosive joint damage of arthritis. The findings suggest that modulation of HIF-1α as a 'master switch' may be used as a therapeutic target in the anti-inflammatory treatment of RA.

## Abbreviations

1D: one dose; 3D: three doses; 3D6d: follow-up at the sixth day after three doses; CFA: complete Freund's adjuvant; HA: hyaluronan; HIF-1α: hypoxia-inducible factor-1-alpha; iNOS: inducible nitric oxide synthase; MMP: matrix metalloproteinase; NO: nitric oxide; NOS: nitric oxide synthases; No-tr: no treatment; OA: osteoarthritis; PBS: phosphate-buffered saline; RA: rheumatoid arthritis; SA: saline; SEM: standard error of the mean; VEGF: vascular endothelial growth factor.

## Competing interests

The authors declare that they have no competing interests.

## Authors' contributions

L-WC conceived of the study, participated in data analysis, and drafted the manuscript. JW participated in the histopathology and scored the immunohistology. P-LC participated in the establishment of the animal model, immunohistology, and animals' functional evaluations. Y-LH conceived of the study, performed the statistical analysis, and drafted the manuscript. All authors read and approved the final manuscript.
